# Acute Angle of Multilobulated Contours Improves the Risk Classification of Thymomas

**DOI:** 10.3389/fmed.2021.744587

**Published:** 2021-09-29

**Authors:** Xiaowei Han, Song Luo, Bing Liu, Yue Chen, Wenwen Gao, Yige Wang, Xiuxiu Liu, Hongwei Yu, Longjiang Zhang, Guolin Ma

**Affiliations:** ^1^Department of Radiology, The Affiliated Drum Tower Hospital of Nanjing University Medical School, Nanjing, China; ^2^Department of Medical Imaging, Jinling Hospital, Medical School of Nanjing University, Nanjing, China; ^3^Department of Radiology, China-Japan Friendship Hospital, Beijing, China

**Keywords:** thymoma, computed tomography, risk classification, predictive model, nomogram

## Abstract

**Background:** Computed tomography plays an important role in the identification and characterization of thymomas. It has been mainly used during preoperative evaluation for clinical staging. However, the reliable prediction of histological risk types of thymomas based on CT imaging features requires further study. In this study, we developed and validated a nomogram based on CT imaging and included new indices for individualized preoperative prediction of the risk classification of thymomas.

**Methods:** We conducted a retrospective, multicenter study that included 229 patients from two Chinese medical centers. All the patients underwent cross-sectional CT imaging within 2 weeks before surgery. The results of pathological assessments were retrieved from existing reports of the excised lesions. The tumor perimeter that contacted the lung (TPCL) was evaluated and a new quantitative indicator, the acute angle (AA) formed by adjacent lobulations, was measured. Two predictive models of risk classification were created using the least absolute shrinkage and selection operator (LASSO) method in a training cohort for features selection. The model with a smaller Akaike information criterion was then used to create an individualized imaging nomogram, which we evaluated regarding its prediction ability and clinical utility.

**Results:** A new CT imaging-based model incorporating AA was developed and validated, which had improved predictive performance during risk classification of thymomas when compared with a model using traditional imaging predictors. The new imaging nomogram with AA demonstrated its clinical utility by decision curve analysis.

**Conclusions:** Acute angle can improve the performance of a CT-based predictive model during the preoperative risk classification of thymomas and should be considered a new imaging marker for the evaluation and treatment of patients with thymomas. On the contrary, TPCL is not useful as a predictor for the risk classification of thymomas in this study.

## Introduction

Thymomas are rare primary thymic epithelial neoplasms, and they account for <1% of all adult malignancies (1). These tumors are often located in the anterior mediastinum and have the potential for local invasion (1, 2). Traditionally, thymomas are usually divided into invasive (Masaoka stage III/IV) and non-invasive (Masaoka stage I/II) lesions according to the Masaoka–Koga clinical staging system (3, 4). Thymomas can also be histologically classified as A, AB, B1, B2, or B3 according to the WHO classification system (revised version of 2015), based on the morphology of epithelial cells and the ratio of lymphocytes to epithelial cells (5). WHO types B2 and B3 are typically considered to be more invasive and are associated with lower survival rates than types A, AB, and B1. Therefore, thymomas can be divided into a low-risk group (types A, AB, and B1) and a high-risk group (types B2 and B3) (6). CT plays an important role in the identification and characterization of thymomas. This imaging technique has been mainly used during preoperative evaluation for clinical staging (4). However, the reliable prediction of histological risk types of thymomas based on CT imaging features still needs further exploration (7, 8).

Previous studies have focused on the relationship between CT imaging findings and the WHO histological classification (9–15). For instance, one study reported that some features (contours, heterogeneous enhancement, infiltration of surrounding fat and lung, and node enlargement) are significantly associated with the WHO classification categories (13). Another study reported that the histological features of aggressive thymomas were significantly correlated with decreased doubling time (DT) and increased growth when DT was evaluated retrospectively and dynamically (14). However, these studies were based exclusively on the evaluation of inter-group CT imaging feature differences and did not include the development of models for classification prediction. Furthermore, other studies have explored the relationship between the tumor perimeter contacting the lung (TPCL) with postoperative pleural recurrence on preoperative CT findings (16, 17). One study developed a more objective and quantitative method, with which the authors measured the angle formed by adjacent lobulations to predict lung invasion by thymomas (18). In this previous study, the authors found that adjacent lung invasion can be precisely predicted by the multilobulated aspect of the thymoma when it includes at least one acute angle (AA). However, the relationship between these quantitative imaging indicators and the WHO histological classification has been rarely reported.

Until now, the classification of thymomas from preoperative CT imaging has mostly employed traditional morphological indicators, without reproducible individualized prediction models or the inclusion of objective and quantitative indicators (19–22). Therefore, in this study, we sought to investigate whether TPCL and an AA formed by adjacent lobulations could constitute quantitative and reliable predictors of thymoma classification. We hypothesized that these new quantitative imaging indicators could be used as independent factors in the development of a predictive model for the risk categories included in the WHO histological classification system of thymomas. Moreover, we also hypothesized that this new model would outperform a model that only includes traditional morphological indicators. The goal was to develop and validate an imaging nomogram to be used in individualized prediction of the risk classifications of thymomas preoperatively using non-invasive data and with minimal demand on patients.

## Methods

### Clinical Samples

**Figure 1:**
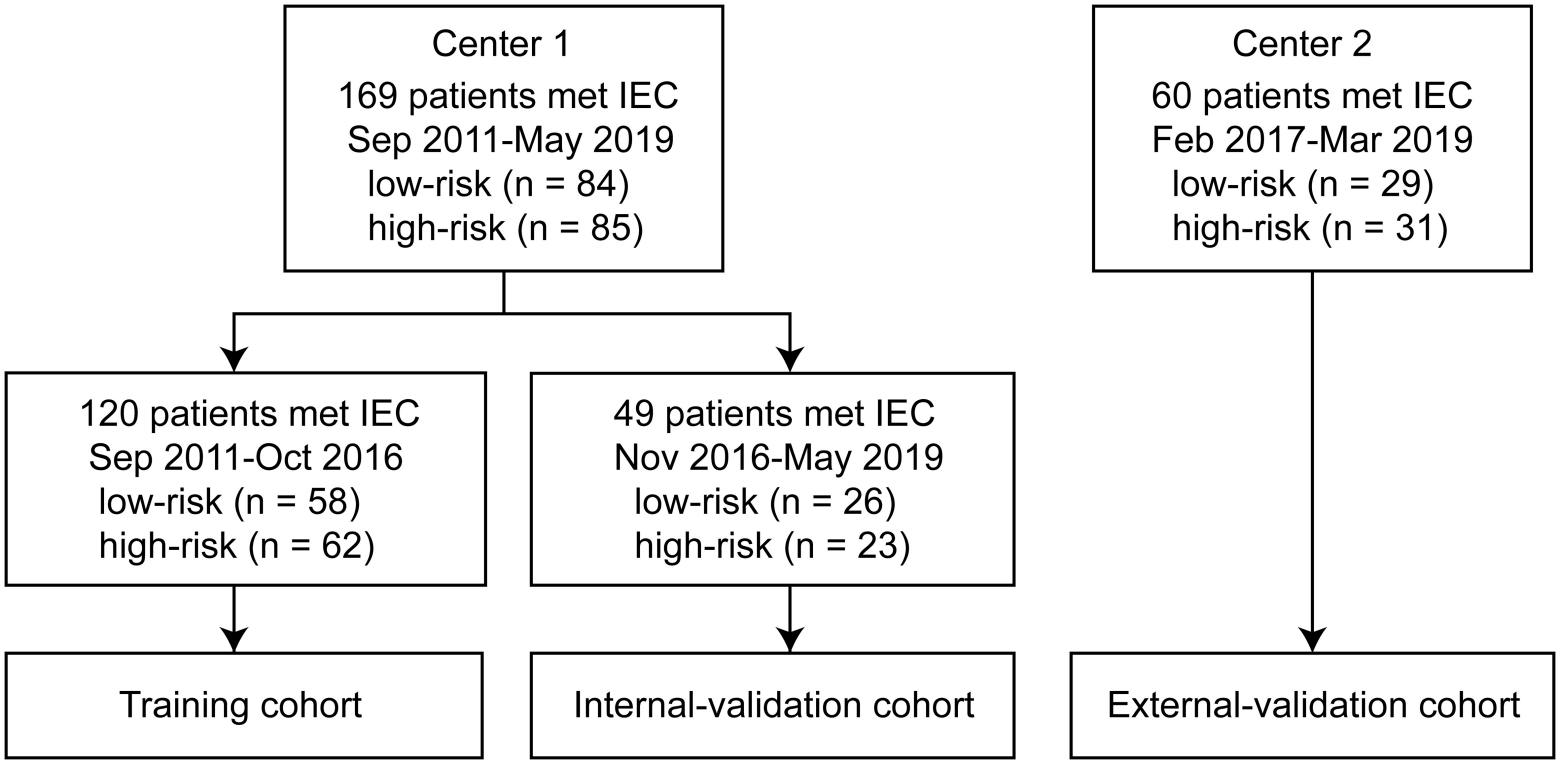
Flow diagram of the study population. The study included 169 patients from center 1 treated between September 2011 and May 2019, and 60 patients from center 2 treated between February 2017 and March 2019. The training cohort included 120 patients (58 low-risk and 62 high-risk) treated consecutively between September 2011 and October 2016 in center 1. The internal validation cohort included 49 patients (26 low-risk and 23 high-risk) treated consecutively between November 2016 and May 2019 in center 1. The external validation cohort included 60 patients (29 low-risk and 31 high-risk) from center 2. IEC, inclusion and exclusion criteria.

We conducted a retrospective multicenter study that included 229 patients. Inclusion criteria were (i) thymoma diagnosed by postoperative pathological examination, (ii) contrast-enhanced CT examination performed, and (iii) CT imaging performed within 2 weeks before surgery. Exclusion criteria were (i) CT imaging performed after preoperative neoadjuvant chemotherapy; (ii) myasthenia gravis, hormone therapy, or other treatment options; (iii) CT artifacts that affected the assessment of the lesions; and (iv) recurrent anterior mediastinal mass after thymectomy. We included 169 patients treated between September 2011 and May 2019 in center 1 and 60 patients treated between February 2017 and March 2019 in center 2. We divided the patients into training, internal validation, and external validation cohorts. The training cohort included 120 patients (58 low-risk and 62 high-risk) treated consecutively between September 2011 and October 2016 in center 1. The internal validation cohort contained 49 patients (26 low-risk and 23 high-risk) treated consecutively between November 2016 and May 2019 in center 1. The external validation cohort included 60 patients (29 low-risk and 31 high-risk) from center 2 (Figure 1). We retrieved related clinical information on age, sex, symptoms, myasthenia gravis, clinical stages, and histological classification from the surgical records and pathological reports in the medical records database. Ethical approval was obtained from the institutional review boards of both center 1 (China-Japan Friendship Hospital in China) and center 2 (Jinling Hospital, Medical School of Nanjing University); the need for informed consent was waived because of the retrospective nature of this study.

### Computed Tomography Imaging

All the patients underwent CT imaging within 2 weeks before surgery and obtained the pathological examination results. They also underwent preoperative cross section spiral CT scanning examinations. CT images were obtained with a variety of scanners, namely, 16-row multi-detector CT (MDCT) (Aquilion; Toshiba, Tokyo, Japan), 320-row MDCT (Aquilion TM ONE; Toshiba, Tokyo, Japan), and 256-row MDCT (Revolution; GE Healthcare, Chicago, IL, United States) in center 1 and dual-source CT (Somatom Definition; Siemens Healthineers, Erlangen, Germany), 128-row MDCT (Somatom Perspective, Siemens Healthineers, Erlangen, Germany), and second-generation dual-source CT (Somatom Flash; Siemens Healthineers, Erlangen, Germany) in center 2. All images were obtained when the patients were in a supine position with suspended inspiration. An intravenously administered contrast medium was used in all the patients. The images were reconstructed from both the mediastinal (window width, 400–450 HU; window level, 20–50 HU) and lung windows (window width, 1,000–1,500 HU; window level, −650 to −750 HU). Images with 5-mm slice thickness after reconstruction were used for evaluation, and original images with 0.6–1.25-mm slice thickness were available, when necessary, in all cases.

### Computed Tomography Imaging Interpretation and Pathological Examination Findings

All the CT images were reviewed using a picture archiving and communications system (PACS; GE Heathcare, Chicago, IL, United States) and were retrospectively reviewed by two radiologists with 10 years of experience, who were all blinded to the clinical details of the patients and pathological findings at the time of image interpretation. Where differences occurred, a third chest tumor radiologist with 23 years of experience addressed the differences for the final decision. The image interpretation criteria used standard reporting terms defined by the International Thymic Malignancy Interest Group (ITMIG) for anterior mediastinal masses suspected to be thymoma (23). Evaluated CT features included the following data about the primary mass and its surrounding structures: lesion location (tumors in the anterior mediastinum were classified into centrally located, right-sided, and left-sided lesions; any tumors that were located around or on the line running through the sternum were considered to be central); size in the x, y, and z axes; contour (smooth, single-lobulated, or irregular multilobulated); internal density (homogenous or heterogeneous); calcifications (without calcification, single, or multiple calcifications); infiltration of surrounding fat; tumor abutment ≥ 50% or <50% of an adjacent mediastinal structure; and direct vascular endoluminal invasion. The following information regarding the surrounding structures was also included: adjacent lung abnormalities, pleural effusion (without, unilateral, or bilateral), mediastinal lymph node enlargement (>1 cm in short axis on an axial image), and phrenic nerve involvement (consistent with elevated hemidiaphragm). Any differences in findings were resolved on a consensual basis.

The thymomas were classified according to the 2015 revised WHO histology classification (5), which is mainly based on the morphology of epithelial cells and the ratio of lymphocytes to epithelial cells. When a tumor showed multiple histological components, it was classified based on the predominant component. The thymomas were divided into low-risk (types A, AB, and B1) and high-risk (types B2 and B3) subgroups, because types B2 and B3 are considered to be more malignant than type A, AB, or B1 (3, 6). A modified Masaoka–Koga stage was obtained by reviewing surgical records and pathological reports (24).

### Measurement of the New Quantitative Indices and Evaluation of Their Consistency

**Figure 2:**
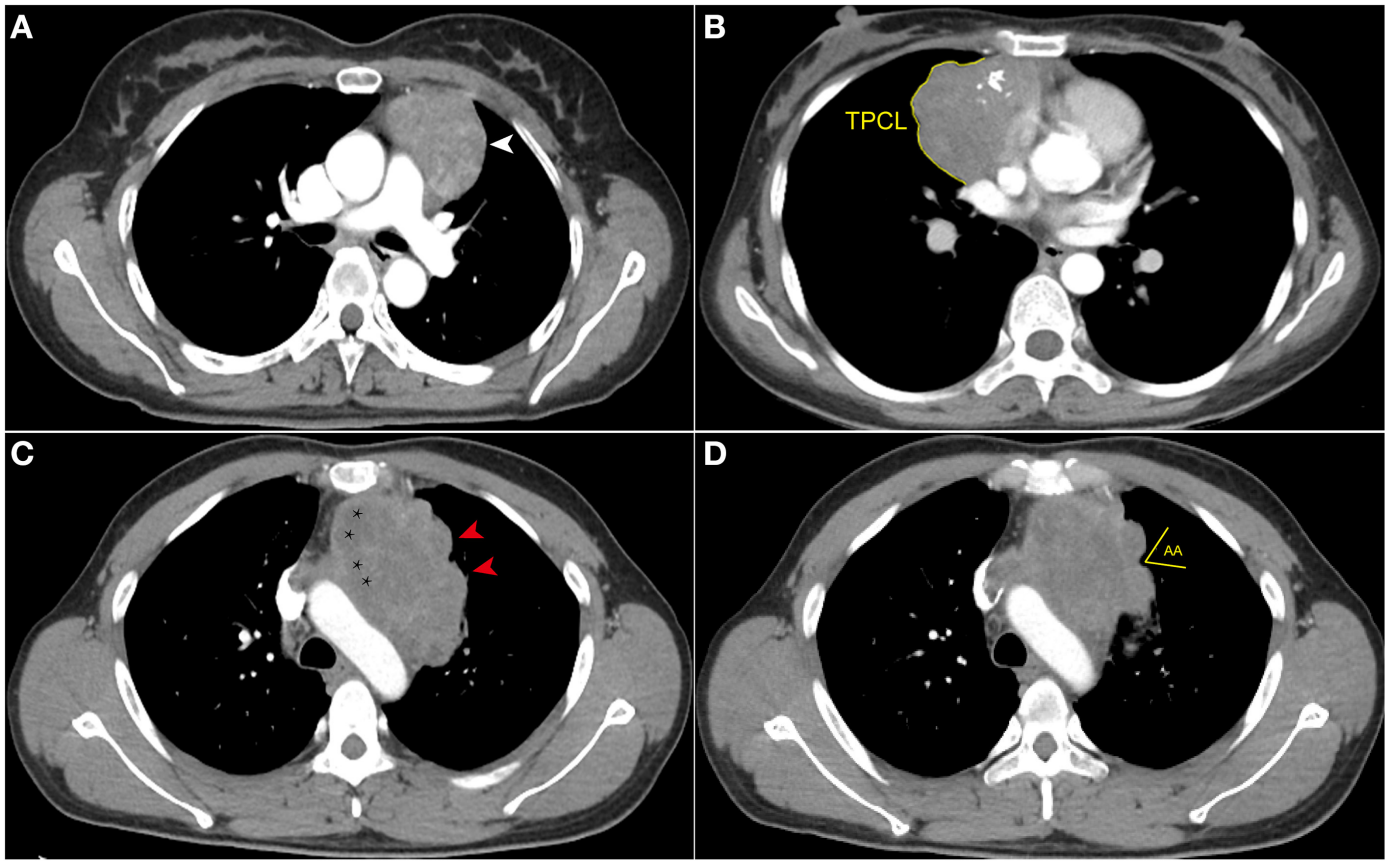
Computed tomography imaging evaluation of contour, internal density, and measurement of the new index. **(A)** Axial contrast-enhanced chest CT image obtained at the level of the pulmonary trunk demonstrated a thymoma with smooth contour (white arrowhead) in a 42-year-old woman (WHO classification AB). **(B)** Axial contrast-enhanced chest CT image showed a thymoma (WHO classification B1) in a 54-year-old woman. The multilobulated contour and internal calcifications were seen, and the tumor perimeter that contacted the lung (TPCL) was measured along the margin between tumor and adjacent lung (yellow arc line). **(C)** Axial contrast-enhanced chest CT image obtained at the level of the aortic arch demonstrated a thymoma (WHO classification B2) in a 49-year-old man who complained of chest distension and chest pain. The tumor has several obtuse angles formed by adjacent lobulations (red arrowhead), and low-density cystic or necrotic areas are indicated by stars. **(D)** In the same patient as **(C)**, the acute angle (AA) formed by adjacent lobulations can be seen in the upper slices.

The maximum tumor diameter was measured at the largest section of the tumor on axial images (size_max) by the same two radiologists who reviewed the CT images. The TPCL was measured by manually drawing the surface of the tumor contour adjacent to the lung, at the two maximum adjacent sections of the tumor shown on axial images. The average TPCL was then calculated as the final value. When the tumor contacted the bilateral mediastinal pleura, only the side overhanging the pleural cavity was measured to measure the TPCL. In multilobulated thymomas interfacing with the lung, the smallest angle formed by adjacent lobulations was uniformly measured twice on lung windows. The average angle was then calculated and classified as an AA or an obtuse angle (OA) (Figure 2). All tumors with smooth contours were counted as OA (18).

Intraclass correlation coefficients were used to determine intra- and inter-observer agreement in the measurement of new indices. A pool of 50 patients was randomly selected from the cohorts, including 25 low-risk and 25 high-risk patients. For inter-observer agreement, two radiologists independently identified the cross-sectional images of the largest tumor area and measured the TPCL and the smallest angle in these patients at the same time. The intraclass correlation coefficients (ICCs) were calculated and analyzed between measurements. To evaluate intra-observer agreement, the TPCL and AA were all measured twice for each patient by each radiologist within 1 month, and the ICCs were separately calculated and analyzed.

### Feature Selection and Building of the Models

For imaging features defined by ITMIG terms and the new quantitative indices, regularized multivariate logistic regression with the least absolute shrinkage and selection operator (LASSO) penalty method was applied to the training cohort to reduce overfitting or any type of bias in feature selection (25). The selected features were then weighted by their respective coefficients in the regression equation formula as follows:


(1) $$\begin{array}{l} y=\beta_{0}+\overset{n}{\underset{s=1}{\sum }}\beta_{i}X_{j}+\varepsilon \end{array}$$

where y is 1 for patients with high-risk thymoma and 0 for low-risk patients, β_0_ is the constant term, n is the number of features used in the model, β_*i*_ (*i* = 0, 1, 2, …, n) is the model parameter of coefficient, *X*_*j*_(*j* = 0, 1, 2, …, n) is the feature, and ε is the error term.

The LASSO criteria for selecting parameters based on minimizing the value of the following cost equation:


(2) $$\begin{array}{l} \overset{N}{\underset{i=1}{\sum }}{\left(y_{i}-\overset{n}{\underset{j=1}{\sum }}X_{ij}\beta_{j}-\beta_{0}\right)}^{2}+\lambda \overset{n}{\underset{j=1}{\sum }}|\beta_{j}| \end{array}$$

where *N* is the number of patients, *y*_*i*_ is the outcome labels of patient i, *n* is the number of features, *X*_*ij*_ is the jth feature of the ith patient, β_*i*_ (*i* = 0, 1, 2, …, n) is the model parameter of coefficient, β_0_ is the constant term, and λ is the regularization parameter.

Least absolute shrinkage and selection operator method is a shrinkage and selection method for linear regression (26). It aims to minimize the sum of squares of residual errors (MSE) under the condition that the sum of absolute values of a regression coefficient that is less than a constant is deleted during variable selection. We selected the optimal value of λ by leave-one-out cross-validation. We considered λ optimal if it minimized MSE and maximized the area under the receiver operating characteristic curve (AUC) in the training cohort. To test the robustness of the final number of features included in the model, we repeated the feature selection procedure at one SE of the optimal λ value (lambda 0.1se).

The model, including the new quantitative index, was compared with a model without this new index using the corresponding Akaike information criterion (AIC) values for each model and their related statistical tests to estimate model complexity and data fitting performance. Furthermore, DeLong tests were performed to compare all pairs of receiver operating characteristic curve (ROC) (27).

### Construction and Evaluation of the Nomogram

We constructed a nomogram based on CT imaging features with the new quantitative index and applied it to predict the risk classification of thymomas. We assessed the accuracy of the nomogram using ROC curves. We then calculated AUCs and compared them between the training cohort and the two validation cohorts by DeLong tests (27). We also determined sensitivity and specificity.

We assessed the calibration of the nomogram using calibration curves and unreliability (U) statistics. We also conducted a decision curve analysis to evaluate the clinical utility of the nomogram by quantifying the net benefit of its use at different threshold probabilities in the validation datasets.

### Statistical Analysis

Statistical analysis was conducted using SPSS (version 23.0; IBM, Armonk, NY, United States), R (version 3.5.0; R Foundation, Vienna, Austria), STATA (version 15.0; StataCorp, College Station TX, United States), and MATLAB (version 2013a; Mathworks, Natick, MA, United States). A two-sided *p* < 0.05 was used as a threshold for statistical significance.

## Results

### Clinical Information and Imaging Features

The clinical characteristics of the patients are summarized in Supplementary Table 1. We found that imaging features, namely, size_max, contour, density, calcification, and the new index, AA, were significantly associated with risk classification in the training cohort (*p* < 0.05). Only AA was significantly associated with risk classification in the internal validation cohort (*p* < 0.05). The inter-group statistical results of features, such as density, calcification, and pleural effusion, approached statistical difference. Size_max, density, and AA were significantly associated with risk classification in the external validation training cohort (*p* < 0.05); contour and calcification trended toward significance.

### Evaluation of the Consistency of the Measurement of the New Quantitative Indices

We found that the measurement of both TPCL and AA presented good inter- and intra-observer agreements. The ICCs were 0.9 for TPCL and 0.85 for AA between the two radiologists. For intra-observer agreement, the ICCs were 0.93 for TPCL and 0.88 for AA in one radiologist, and 0.92 for TPCL and 0.89 for AA in the other.

### Feature Selection and Model Development

**Figure 3:**
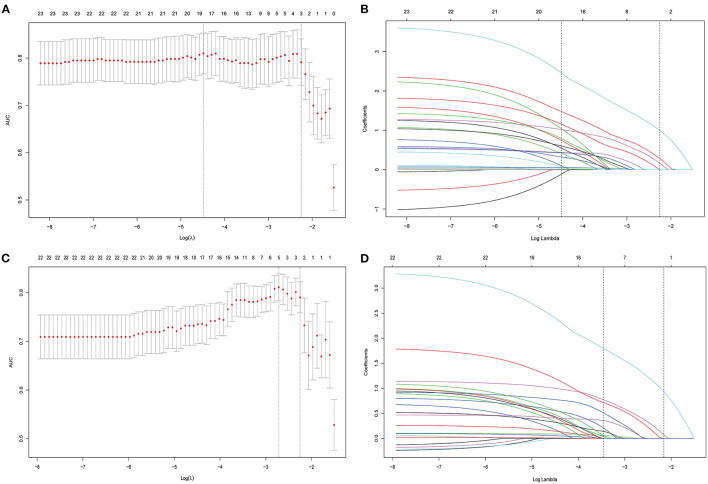
All imaging features were selected by regularized multivariate logistic regression with the least absolute shrinkage and selection operator (LASSO) method in the training cohort. **(A)** Twenty-three features (with AA) were reduced to three potential predictors to build the first model in the training cohort; the area under the receiver operating characteristic curve (AUC) was plotted vs. log (λ). Dotted vertical lines were drawn at the optimal values using the minimum criteria and the 1 standard error of the minimum criteria (the 1-SE criteria). **(B)** A coefficient profile plot was produced against the log (λ) sequence. A vertical line was drawn at the value selected by 10-fold cross-validation, where optimal λ (0.1052) resulted in 3 non-zero coefficients. **(C)** Twenty-two features (without AA) were reduced to two potential predictors to build the second model in the training cohort; the area under the receiver operating characteristic (AUC) curve was plotted vs. log (λ). Dotted vertical lines were drawn at the optimal values using the minimum criteria and the 1 standard error of the minimum criteria (the 1-SE criteria). **(D)** A coefficient profile plot was produced against the log (λ) sequence. A vertical line was drawn at the value selected by 10-fold cross-validation, where optimal λ (0.1155) resulted in 2 non-zero coefficients.

**Figure 4:**
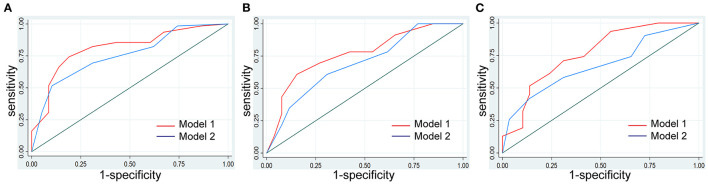
Model development and comparison. The model with AA of multilobulated contour identified as a new independent predictor improved the identification performance for risk classification of thymoma compared with the model without AA. **(A)** Comparison of the performance of the models in the training cohort. **(B)** Comparison of the performance of the models in the internal validation cohort. **(C)** Comparison of the performance of the models in the external validation cohort.

From all the imaging features, 23 (with AA) were reduced to three potential predictors based on data from the 120 patients of the training cohort (8:1 ratio; Figures 3A,B). These three features were retained with non-zero coefficients in the LASSO logistic regression model with a minimum λ of 0.1052 and then used in the regression equation to build the first model. The result demonstrated that TPCL was ineligible for the risk classification of thymoma (11:1 ratio; Figure 3). Without considering the AA factor, 22 features were reduced to two predictors with a minimum λ of 0.1155 based on data from the training cohort. These two features were used in the regression equation to build the second model (Supplementary Table 2). We found significant differences between the two models for both AIC and AUC (Supplementary Table 3; Figure 4).

### Construction and Evaluation of an Individualized Nomogram

**Figure 5:**
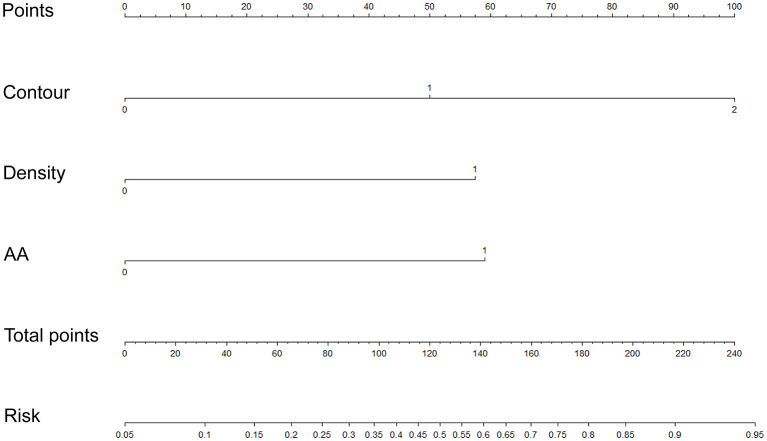
Construction of an individualized imaging nomogram in the training cohort incorporated three independent predictors consisting of contour, density, and AA.

We used the first model with the smaller AIC to construct an individualized imaging nomogram incorporating three independent predictors: contour, density, and AA (Figure 5). The classification accuracies were 77.5, 73.47, and 70% in the training, internal validation, and external validation cohorts, respectively. The sensitivities were 74.19, 60.87, and 70.97%, and the specificities were 81.03, 84.62, and 68.97% in the training, internal validation, and external validation cohorts, respectively (Supplementary Table 4; Figure 4).

### Validation of Individualized Nomogram

We found that calibrations for the probability of risk classification of thymomas were good in the three cohorts (training, *p* = 0.062; internal validation, *p* = 0.267; external validation, *p* = 0.14). The C-index of the nomogram for the prediction of lymph node status was 0.811 (95% CI, 0.731 to 0.889), 0.766 (95% CI, 0.63 to 0.902), and 0.765 (95% CI, 0.644 to 0.886) in the training, internal validation, and external validation cohorts, respectively (Supplementary Table 2; Supplementary Figure 1).

### Clinical Use

**Figure 6:**
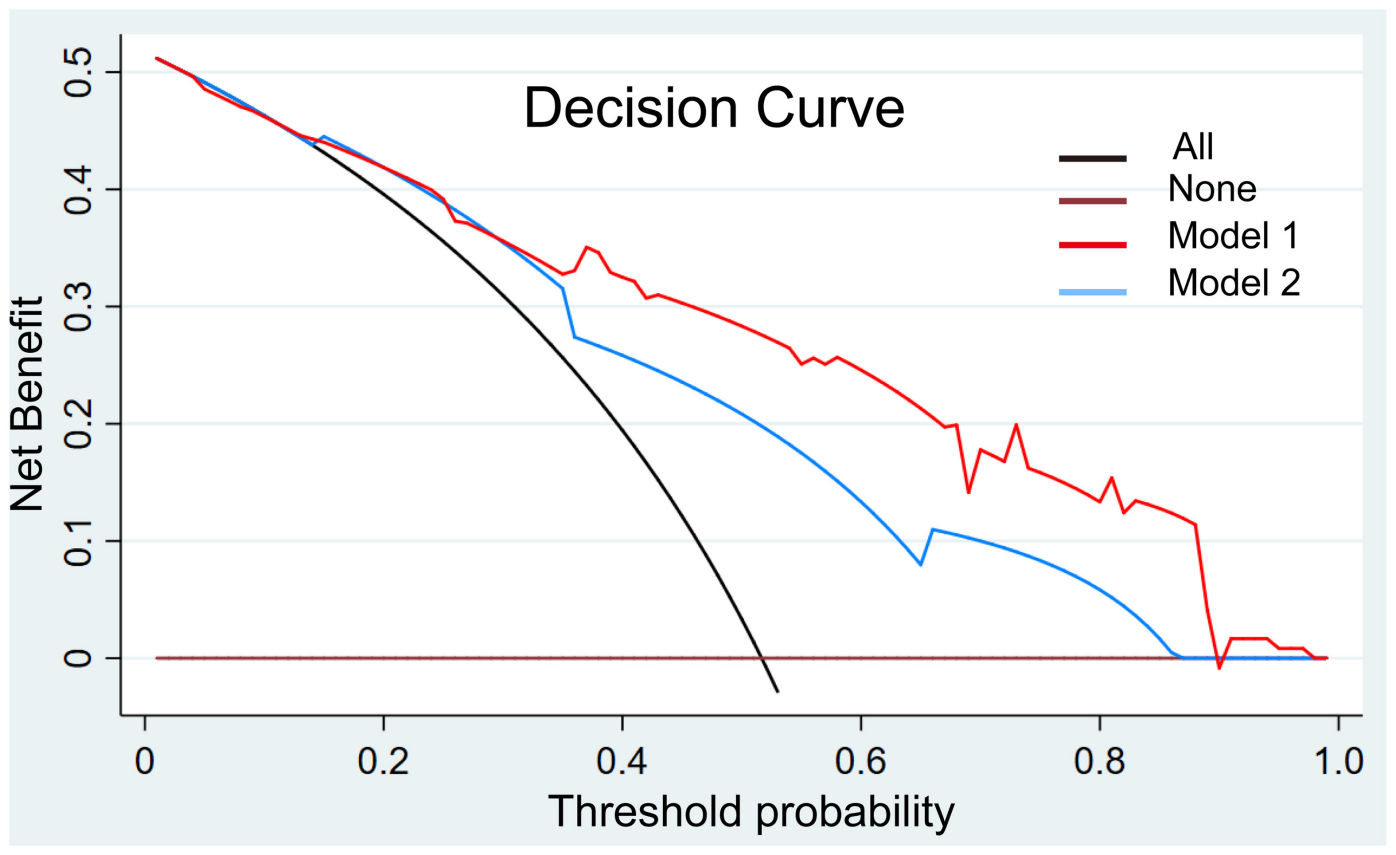
Decision curve analysis with the comparison of the imaging models. The decision curve showed that if the threshold probability in the clinical decision is more than 18%, using the imaging nomogram to predict risk classification adds more benefit than either the treat-all-patients scheme or the treat-none scheme. Within this range, the net benefit was comparable based on two models with several overlaps, and the model with AA showed better.

We present the decision curve analysis for the first and second models in Figure 6. The decision curve showed that if the threshold probability in the clinical decision is higher than 18%, using the radiomics nomogram to predict risk classification performs better than either the treat-all-patients or the treat-none schemes. Within this range, the net benefit was comparable between the two models with several overlaps.

## Discussion

In this study, we developed and validated a diagnostic CT-based predictive model for individualized preoperative risk classification in patients with thymomas. The new model incorporates contour, density, and the new quantitative index AA. The new model successfully stratified the histological grading of tumors according to their risk classifications. We established a nomogram that can facilitate the clinical evaluation and treatment of thymomas.

In this study, all the patients underwent preoperative cross-sectional spiral CT examinations using the same body position and scanning methods. However, we should highlight that the CT images were obtained with different types of scanners in the two centers. This is a common limitation in multicenter studies, and we have performed the following measures to improve the consistency of the results. First, all the patients underwent preoperative cross-section spiral CT examinations. They were scanned in the same body position and same methods, although the CT images were obtained with different types of scanners. All the images were reconstructed with the same parameters and methods, thus ensuring consistent evaluation and measurement of image quality and reducing errors in imaging processes among the different CT scanners (28, 29). In addition, the research focused on the imaging features and quantified indicators based on CT from the perspective of clinical diagnosis. Therefore, any error mainly comes from the experience of the radiologists and measurements. We introduced two new key quantitative indices, TPCL and AA (16–18) and showed that they can be consistently measured. Indeed, the results of the consistency analysis by the ICC evaluation of inter- and intra-observer agreements showed that the consistencies of both TPCL and AA measurements were satisfactory. The use of standardized techniques in this study minimized errors related to image assessment, assuring the quality of these new quantitative indices (30).

During the construction of the first and second models in this study, the candidate imaging features were reduced to three or two potential predictors using the LASSO method to obtain a subset of features with optimal stability and accuracy (25, 26). The LASSO regression algorithm is a regularized feature selection method with which all independent variables are processed simultaneously based on variance trade-off. Several previous studies have demonstrated the advantages of using LASSO to select parameters when constructing predictive models (25, 26, 31, 32). However, we are not aware of any study that has used this method for histological grading of thymomas. Tumor size, contours, internal density, calcification, and the new index AA all showed significant differences between low- and high-risk thymomas in this study. However, tumor size, TPCL, and calcification were not selected as independent risk factors in the final regression model. These results match those of previous studies in the field, although we should note that CT imaging was shown to not differentiate between histological subtypes of thymomas in these previous studies. Of note, Johnson et al. reported an association between CT imaging and pathologic assessments of the tumor response after neoadjuvant treatment (21). Tumor size and calcification have been found to be related to type B3 thymoma in previous studies; however, we could not replicate these findings (33).

By comparing AICs and AUCs between the two models, we show that the new model that included AA has better predictive performance. The AIC is a standard method for estimating associations between model complexity and data-fitting performance (34). In this study, we used the AIC to examine whether the inclusion of three predictors in the first model would counteract the performance benefits by bringing additional complexity costs. We found that this is not the case. In fact, the differences between the two models in the AIC pinpointed that the benefits of including an extra predictor outweighed the costs caused by the increased complexity. We also compared the predictive performance of the two models by testing for differences between their AUCs by DeLong tests. We found that the new model with AA performed better in the identification of risk classifications in thymomas in all the three cohorts, but we determined that TPCL is ineligible for risk classification of thymoma. The presentation of an AA in tumors indicates a high-risk pathological type with high invasion potential. Therefore, we speculate that AA, being an independent predictor of risk category, is closely related to this pathological category of thymomas (18).

Thymomas are heterogeneous tumors, and their histopathology grading is associated with multiple imaging features (8, 35–37). Choe et al. reported that the histological features of aggressive tumors were significantly correlated with decreased DT and increased growth (14). In addition, Green et al. found that the presence of an AA between lobulations in multilobulated thymomas can predict lung invasion with satisfactory accuracy (18). Until now, there has been no study reporting the reliable identification of histological types of thymomas based on CT imaging features (36). We aimed to bridge this gap by constructing and validating a nomogram incorporating new quantitative imaging variables. The nomogram presents a good performance in generating individualized probabilities of the risk classification of thymomas. The results demonstrated good generalizability and provided a tool that can be used by both physicians and patients to perform preoperative individualized prediction of the risk classification of thymomas, following the current trends toward personalized medicine.

The introduction of the model in clinical practice has the potential to benefit both the diagnosis and treatment of patients with thymomas. We comprehensively evaluated the discrimination and calibration of the risk classification predictions and demonstrate the reliability of the results of the model. However, it is also important to consider whether the nomogram-assisted decisions would improve clinical outcomes. We performed a decision curve analysis to examine the clinical consequences of the tool based on different threshold probabilities and net benefits. We implemented this analysis by calculating the proportion of true positives minus the proportion of false positives weighted by the relative harm of false-positive and false-negative results. We found that for threshold probabilities higher than 18%, the use of the nomogram to predict risk classification outperforms both the treat-all-patients and the treat-none schemes. This analysis demonstrates the clinical utility of the tool.

This study has some limitations. First, the incidence rate of thymoma was very low. It is very precious and time-consuming to obtain patients with complete clinical, imaging (qualified contrast- enhanced CT), and definite pathology results. Second, we relied on data collected retrospectively; 120 and 49 patients were used for model training and internal validation in this study, respectively, while only 60 patients were included for external validation. Compared with the training set and internal validation set, the proportion of the two risk types was relatively balanced and may support the results of this study. Third, the CT scanners used in this study and their parameters were different, and the diaphragm evaluation was based on the coronal reconstruction of images. However, we suggest that these parameters are unlikely to have strongly affected the evaluation of parameters that can be assessed and measured using CT images. Using high-dimensional data from a quantitative analysis of tumor volume and radiomics could improve the predictive models.

## Conclusions

In conclusion, we developed and validated a new CT-based model incorporating AA that can improve predictive performance during the risk classification of thymomas, when compared with traditional imaging predictors. By decision curve analysis, we demonstrated the clinical utility of the tool. AA should be considered as a new imaging marker for the evaluation and treatment of patients with thymomas.

## Data Availability Statement

The original contributions presented in the study are included in the article/Supplementary Material, further inquiries can be directed to the corresponding author/s.

## Ethics Statement

The authors are accountable for all aspects of the work in ensuring that questions related to the accuracy or integrity of any part of the work are appropriately investigated and resolved. Ethical approval was obtained from the institutional review boards and the informed consents were waived from the patients for the retrospective nature of this study.

## Author Contributions

XH and SL acquired, analyzed, explained the data, and drafted the manuscript. HY, YC, BL, and WG analyzed and explained the imaging data. YW and XL acquired the clinical information and revised the manuscript. LZ and GM designed the study and revised the manuscript. All authors contributed to the article and approved the submitted version.

## Funding

This research was funded by Beijing science and technology planning project (Z211100003521009).

## Conflict of Interest

The authors declare that the research was conducted in the absence of any commercial or financial relationships that could be construed as a potential conflict of interest.

## Publisher's Note

All claims expressed in this article are solely those of the authors and do not necessarily represent those of their affiliated organizations, or those of the publisher, the editors and the reviewers. Any product that may be evaluated in this article, or claim that may be made by its manufacturer, is not guaranteed or endorsed by the publisher.

## Supplementary Material

Supplementary Figure 1 Calibration curves of the imaging nomogram in the **(A)** training cohort, **(B)** internal validation cohort, and **(C)** external validation cohorts. The y-axis represents the actual results of risk classifications. The x-axis represents the predicted results. The diagonal gray line represents a perfect prediction by an ideal model. The fit double arcs represent allowable errors in unreliability (*U*) statistic. The dotted line represents the performance of the nomogram, of which a closer fit to the arc line represents a better prediction. Good calibrations were observed for the probability of risk classification of thymomas in the training (*P* = 0.062), internal validation (*P* = 0.267), and external validation cohorts (*P* = 0.14).

Supplementary Table 1 Clinical information and imaging features of patients in the training and validation cohorts.

Supplementary Table 2 Risk factors for imaging features models.

Supplementary Table 3 Evaluation of the imaging features models among cohorts.

Supplementary Table 4 Evaluation of individualized nomogram among cohorts.

## References

[1] Marom EM. Advances in thymoma imaging. J Thorac Imaging. (2013) 28:69–80. 10.1097/RTI.0b013e31828609a0 23422781

[2] Marom EM. Imaging thymoma. J Thorac Oncol. (2010) 5(10Suppl.4):S296–303. 10.1097/JTO.0b013e3181f209ca 20859123

[3] Klimiec E Quirke M Leite MI Hilton-Jones D. Thymus imaging in myasthenia gravis: the relevance in clinical practice. Muscle Nerve. (2018) 9:26096. 10.1002/mus.26096 29424940

[4] Carter BW Benveniste MF Madan R Godoy MC Groot PM Truong MT. IASLC/ITMIG staging system and lymph node map for thymic epithelial neoplasms. Radiographics. (2017) 37:758–76. 10.1148/rg.2017160096 28493800

[5] Marx A Chan JK Coindre JM Detterbeck F Girard N Harris NL. The 2015 world health organization classification of tumors of the thymus: continuity and changes. J Thorac Oncol. (2015) 10:1383–95. 10.1097/JTO.0000000000000654 26295375 PMC4581965

[6] Jeong YJ Lee KS Kim J Shim YM Han J Kwon OJ. Does CT of thymic epithelial tumors enable us to differentiate histologic subtypes and predict prognosis? Am J Roentgenol. (2004) 183:283–9. 10.2214/ajr.183.2.1830283 15269013

[7] Priola AM Priola SM Giraudo MT Gned D Fornari A Ferrero B. Diffusion-weighted magnetic resonance imaging of thymoma: ability of the Apparent Diffusion Coefficient in predicting the World Health Organization (WHO) classification and the Masaoka-Koga staging system and its prognostic significance on disease-free survival. Eur Radiol. (2016) 26:2126–38. 10.1007/s00330-015-4031-6 26427698

[8] Padda SK Terrone D Tian L Khuong A Neal JW Riess JW. Computed tomography features associated with the eighth edition TNM stage classification for thymic epithelial tumors. J Thorac Imaging. (2018) 33:176–83. 10.1097/RTI.0000000000000310 29219888 PMC6368176

[9] Yanagawa M Tomiyama N. Prediction of thymoma histology and stage by radiographic criteria. Thorac Surg Clin. (2011) 21:1–12. 10.1016/j.thorsurg.2010.08.008 21070982

[10] Shen Y Ye J Fang W Zhang Y Ye X Ma Y. Efficacy of computed tomography features in predicting stage III thymic tumors. Oncol Lett. (2017) 13:29–36. 10.3892/ol.2016.5429 28123518 PMC5245091

[11] Hayes SA Huang J Golia Pernicka J Cunningham J Zheng J Moskowitz CS. Radiographic predictors of resectability in thymic carcinoma. Ann Thorac Surg. (2018) 106:242–8. 10.1016/j.athoracsur.2018.02.019 29534953 PMC6853178

[12] Hayes SA Huang J Plodkowski AJ Katzen J Zheng J Moskowitz CS. Preoperative computed tomography findings predict surgical resectability of thymoma. J Thorac Oncol. (2014) 9:1023–30. 10.1097/JTO.0000000000000204 24926547

[13] Zhao Y Chen H Shi J Fan L Hu D Zhao H. The correlation of morphological features of chest computed tomographic scans with clinical characteristics of thymoma. Eur J Cardiothorac Surg. (2015) 48:698–704. 10.1093/ejcts/ezu475 25527169

[14] Choe J Lee SM Lim S Choi SH Kim N Do KH. Doubling time of thymic epithelial tumours on CT: correlation with histological subtype. Eur Radiol. (2017) 27:4030–6. 10.1007/s00330-017-4795-y 28332015

[15] Luo H Xie S Ma C Zhang W Tschöpe C Fa X. Correlation between thymus radiology and myasthenia gravis in clinical practice. Front Neurol. (2019) 9:1173. 10.3389/fneur.2018.01173 30697185 PMC6340958

[16] Kato T Iwano S Taniguchi T Kawaguchi K Fukui T Ishiguro F. The contact length between the tumor contour and the lung on computed tomography is a risk factor for pleural recurrence after complete resection of thymoma. Gen Thorac Cardiovasc Surg. (2015) 63:343–8. 10.1007/s11748-015-0525-z 25663292

[17] Do YW Lee HJ Narm KS Jung HS Lee JG Kim DJ. Tumor perimeter and lobulation as predictors of pleural recurrence in patients with resected thymoma. Lung Cancer. (2016) 98:79–83. 10.1016/j.lungcan.2016.05.017 27393511

[18] Green DB Eliades S Legasto AC Askin G Port JL Gruden JF. Multilobulated thymoma with an acute angle: a new predictor of lung invasion. Eur Radiol. (2019) 29:4555–62. 10.1007/s00330-019-06059-1 30809718

[19] Ackman JB Verzosa S Kovach AE Louissaint A Jr Lanuti M Wright CD. High rate of unnecessary thymectomy and its cause. Can computed tomography distinguish thymoma, lymphoma, thymic hyperplasia, and thymic cysts? Eur J Radiol. (2015) 84:524–33. 10.1016/j.ejrad.2014.11.042 25575742

[20] Khandelwal A Sholl LM Araki T Ramaiya NH Hatabu H Nishino M. Patterns of metastasis and recurrence in thymic epithelial tumours: longitudinal imaging review in correlation with histological subtypes. Clin Radiol. (2016) 71:1010–7. 10.1016/j.crad.2016.05.007 27267746 PMC5010483

[21] Johnson GB Aubry MC Yi ES Koo CW Jenkins SM Garces YI. Radiologic response to neoadjuvant treatment predicts histologic response in thymic epithelial tumors. J Thorac Oncol. (2017) 12:354–67. 10.1016/j.jtho.2016.09.118 27666658

[22] Okumura M Yoshino I Yano M Watanabe SI Tsuboi M Yoshida K. Tumour size determines both recurrence-free survival and disease-specific survival after surgical treatment for thymoma. Eur J Cardiothorac Surg. (2019) 56:174–81. 10.1093/ejcts/ezz001 30783650

[23] Marom EM Rosado-de-Christenson ML Bruzzi JF Hara M Sonett JR Ketai L. Standard report terms for chest computed tomography reports of anterior mediastinal masses suspicious for thymoma. J Thorac Oncol. (2011) 6:S1717–23. 10.1097/JTO.0b013e31821e8cd6 21847053

[24] Detterbeck FC Nicholson AG Kondo K Van Schil P Moran C. The Masaoka-Koga stage classification for thymic malignancies: clarification and definition of terms. J Thorac Oncol. (2011) 6:S1710–6. 10.1097/JTO.0b013e31821e8cff 21847052

[25] Huang YQ Liang CH He L Tian J Liang CS Chen X. Development and validation of a radiomics nomogram for preoperative prediction of lymph node metastasis in colorectal cancer. J Clin Oncol. (2016) 34:2157–64. 10.1200/JCO.2015.65.9128 27138577

[26] Dong D Tang L Li ZY Fang MJ Gao JB Shan XH. Development and validation of an individualized nomogram to identify occult peritoneal metastasis in patients with advanced gastric cancer. Ann Oncol. (2019) 30:431–8. 10.1093/annonc/mdz001 30689702 PMC6442651

[27] Moons KG Altman DG Reitsma JB Ioannidis JP Macaskill P Steyerberg EW. Transparent reporting of a multivariable prediction model for Individual Prognosis or Diagnosis (TRIPOD): explanation and elaboration. Ann Intern Med. (2015) 162:W1–73. 10.7326/M14-0698 25560730

[28] Dong D Fang MJ Tang L Shan XH Gao JB Giganti F. Deep learning radiomic nomogram can predict the number of lymph node metastasis in locally advanced gastric cancer: an international multicenter study. Ann Oncol. (2020) 31:912–20. 10.1016/j.annonc.2020.04.003 32304748

[29] Wang S Shi J Ye Z Dong D Yu D Zhou M. Predicting EGFR mutation status in lung adenocarcinoma on computed tomography image using deep learning. Eur Respir J. (2019) 53:1800986. 10.1183/13993003.00986-2018 30635290 PMC6437603

[30] Song J Shi J Dong D Fang M Zhong W Wang K. A new approach to predict progression-free survival in stage IV EGFR-mutant NSCLC patients with EGFR-TKI therapy. Clin Cancer Res. (2018) 24:3583–92. 10.1158/1078-0432.CCR-17-2507 29563137

[31] Liu Z Zhang XY Shi YJ Wang L Zhu HT Tang Z. Radiomics analysis for evaluation of pathological complete response to neoadjuvant chemoradiotherapy in locally advanced rectal cancer. Clin Cancer Res. (2017) 23:7253–62. 10.1158/1078-0432.CCR-17-1038 28939744

[32] Wang X Zhao X Li Q Xia W Peng Z Zhang R. Can peritumoral radiomics increase the efficiency of the prediction for lymph node metastasis in clinical stage T1 lung adenocarcinoma on CT? Eur Radiol. (2019) 29:6049–58. 10.1007/s00330-019-06084-0 30887209

[33] Han X Gao W Chen Y Du L Duan J Yu H. Relationship between computed tomography imaging features and clinical characteristics, Masaoka-Koga Stages, and World Health Organization histological classifications of thymoma. Front Oncol. (2019) 9:1041. 10.3389/fonc.2019.01041 31681579 PMC6798238

[34] Coutant C Olivier C Lambaudie E Fondrinier E Marchal F Guillemin F. Comparison of models to predict nonsentinel lymph node status in breast cancer patients with metastatic sentinel lymph nodes: a prospective multicenter study. J Clin Oncol. (2009) 27:2800–8. 10.1200/JCO.2008.19.7418 19349546

[35] Pandey S Jaipal U Mannan N Yadav R. Diagnostic accuracy of multidetector computed tomography scan in mediastinal masses assuming histopathological findings as gold standard. Pol J Radiol. (2018) 83:e234–42. 10.5114/pjr.2018.76709 30627241 PMC6323600

[36] Carter BW Benveniste MF Truong MT Marom EM. State of the art: MR imaging of thymoma. Magn Reson Imaging Clin N Am. (2015) 23:165–77. 10.1016/j.mric.2015.01.005 25952513

[37] Hwang Y Park IK Park S Kim ER Kang CH Kim YT. Lymph node dissection in thymic malignancies: implication of the ITMIG lymph node map, TNM stage classification, and recommendations. J Thorac Oncol. (2016) 11:108–14. 10.1016/j.jtho.2015.09.001 26762745

