# Supplementary material for: Acute Angle of Multilobulated Contours Improves the Risk Classification of Thymomas
Source: Front Med (Lausanne). 2021 Sep 29;8:744587. doi: 10.3389/fmed.2021.744587 (PMC8513789; doi:10.3389/fmed.2021.744587)
Supplement: Supplementary Table 1 — Clinical information and imaging features of patients in the training and validation cohorts. [file Table_1.docx]

**Acute angle of multilobulated contour improves risk classification of thymomas**

**Supplementary** **Table S1**. Clinical information and imaging features of patients in the training and validation cohorts

|  | **Training cohort** | |  | **Internal-validation cohort** | |  | **External-validation cohort** | |  |
| --- | --- | --- | --- | --- | --- | --- | --- | --- | --- |
| Factors | low-risk | high-risk | p-value | low-risk | high-risk | p-value | low-risk | high-risk | p-value |
| Sex |  |  | 0.37 |  |  | 0.87 |  |  | 0.80 |
| male | 32 (55%) | 29 (47%) |  | 13 (50%) | 12 (52%) |  | 14 (48%) | 13 (42%) |  |
| female | 26 (45%) | 33 (53%) |  | 13 (50%) | 11 (48%) |  | 15 (52%) | 18 (58%) |  |
| Age, mean (SD), years | 48.03 (14.73) | 48.79 (14.98) | 0.78 | 48.12 (15.71) | 47.35 (16.17) | 0.87 | 48.07 (14.71) | 47.71 (16.08) | 0.93 |
| Size_max, mean (SD), cm | 6.07 (1.82) | 6.94 (2.24) | 0.023* | 6.24 (2.08) | 6.58 (1.84) | 0.55 | 6.19 (1.81) | 6.85 (2.20) | 0.041* |
| Location |  |  | 0.85 |  |  | 0.72 |  |  | 0.95 |
| centrally | 20 (34%) | 18 (29%) |  | 9 (35%) | 8 (35%) |  | 10 (34%) | 12 (39%) |  |
| left-sided | 22 (38%) | 26 (42%) |  | 9 (35%) | 10 (43%) |  | 11 (38%) | 12 (39%) |  |
| right-sided | 16 (28%) | 18 (29%) |  | 8 (31%) | 5 (22%) |  | 8 (28%) | 7 (23%) |  |
| Contour |  |  | <0.001* |  |  | 0.14 |  |  | 0.057* |
| smooth | 22 (38%) | 11 (18%) |  | 10 (38%) | 5 (22%) |  | 10 (34%) | 8 (26%) |  |
| single lobulated | 30 (52%) | 19 (31%) |  | 13 (50%) | 10 (43%) |  | 15 (52%) | 10 (32%) |  |
| multi-lobulated | 6 (10%) | 32 (52%) |  | 3 (12%) | 8 (35%) |  | 4 (14%) | 13 (42%) |  |
| Density |  |  | 0.006* |  |  | 0.085* |  |  | 0.021* |
| homogenous | 36 (62%) | 22 (35%) |  | 15 (58%) | 7 (30%) |  | 21 (72%) | 13 (42%) |  |
| heterogeneous | 22 (38%) | 40 (65%) |  | 11 (42%) | 16 (70%) |  | 8 (28%) | 18 (58%) |  |
| Calcification |  |  | 0.016* |  |  | 0.091* |  |  | 0.073* |
| without calcification | 41 (71%) | 30 (48%) |  | 18 (69%) | 9 (39%) |  | 20 (69%) | 16 (52%) |  |
| single calcification | 10 (17%) | 12 (19%) |  | 5 (19%) | 7 (30%) |  | 6 (21%) | 4 (13%) |  |
| multiple calcifications | 7 (12%) | 20 (32%) |  | 3 (12%) | 7 (30%) |  | 3 (10%) | 11 (35%) |  |
| Infiltration of surrounding fat |  |  | 0.72 |  |  | 0.26 |  |  | 0.45 |
| no | 34 (59%) | 34 (55%) |  | 16 (62%) | 10 (43%) |  | 17 (59%) | 15 (48%) |  |
| yes | 24 (41%) | 28 (45%) |  | 10 (38%) | 13 (57%) |  | 12 (41%) | 16 (52%) |  |
| Tumor abutting≥50% |  |  | 0.22 |  |  | 0.15 |  |  | 0.26 |
| no | 45 (78%) | 41 (66%) |  | 18 (69%) | 11 (48%) |  | 23 (79%) | 20 (65%) |  |
| yes | 13 (22%) | 21 (34%) |  | 8 (31%) | 12 (52%) |  | 6 (21%) | 11 (35%) |  |
| Tumor abutting<50% |  |  | 0.86 |  |  | 0.15 |  |  | 0.20 |
| no | 30 (52%) | 30 (48%) |  | 15 (58%) | 8 (35%) |  | 19 (66%) | 15 (48%) |  |
| yes | 28 (48%) | 32 (52%) |  | 11 (42%) | 15 (65%) |  | 10 (34%) | 16 (52%) |  |
| Vascular invasion |  |  | 0.11 |  |  | 0.18 |  |  | 0.30 |
| no | 50 (86%) | 46 (74%) |  | 22 (85%) | 15 (65%) |  | 26 (90%) | 24 (77%) |  |
| yes | 8 (14%) | 16 (26%) |  | 4 (15%) | 8 (35%) |  | 3 (10%) | 7 (23%) |  |
| Abnormal adjacent lung |  |  | 0.46 |  |  | 0.39 |  |  | 0.31 |
| no | 36 (62%) | 34 (55%) |  | 17 (65%) | 12 (52%) |  | 18 (62%) | 15 (48%) |  |
| yes | 22 (38%) | 28 (45%) |  | 9 (35%) | 11 (48%) |  | 11 (38%) | 16 (52%) |  |
| Pleural effusion |  |  | 0.33 |  |  | 0.059* |  |  | 0.14 |
| without | 44 (76%) | 39 (63%) |  | 20 (77%) | 10 (43%) |  | 22 (76%) | 16 (52%) |  |
| unilateral | 10 (17%) | 16 (26%) |  | 5 (19%) | 10 (43%) |  | 5 (17%) | 12 (39%) |  |
| bilateral | 4 (7%) | 7 (11%) |  | 1 (4%) | 3 (13%) |  | 2 (7%) | 3 (10%) |  |
| Lymph node |  |  | 0.40 |  |  | 0.17 |  |  | 0.79 |
| without enlargement | 53 (91%) | 53 (85%) |  | 25 (96%) | 19 (83%) |  | 26 (90%) | 27 (87%) |  |
| enlargement | 5 (9%) | 9 (15%) |  | 1 (4%) | 4 (17%) |  | 3 (10%) | 4 (13%) |  |
| Elevated hemidiaphragm |  |  | 0.82 |  |  | 0.48 |  |  | 0.50 |
| no | 47 (81%) | 49 (79%) |  | 22 (85%) | 17 (74%) |  | 23 (79%) | 27 (87%) |  |
| yes | 11 (19%) | 13 (21%) |  | 4 (15%) | 6 (26%) |  | 6 (21%) | 4 (13%) |  |
| Symtom |  |  | 0.57 |  |  | 0.40 |  |  | 0.29 |
| no | 39 (67%) | 38 (61%) |  | 16 (62%) | 11 (48%) |  | 21 (72%) | 18 (58%) |  |
| yes | 19 (33%) | 24 (39%) |  | 10 (38%) | 12 (52%) |  | 8 (28%) | 13 (42%) |  |
| Myasthenia gravis |  |  | 0.61 |  |  | 0.12 |  |  | 0.73 |
| no | 51 (88%) | 52 (84%) |  | 24 (92%) | 17 (74%) |  | 25 (86%) | 25 (81%) |  |
| yes | 7 (12%) | 10 (16%) |  | 2 (8%) | 6 (26%) |  | 4 (14%) | 6 (19%) |  |
| TPCL, mean (SD), cm | 4.81 (2.28) | 4.96 (2.31) | 0.72 | 4.70 (1.83) | 5.06 (2.21) | 0.54 | 4.66 (2.16) | 5.13 (2.48) | 0.44 |
| AA |  |  | 0.002* |  |  | 0.047* |  |  | 0.021* |
| no | 36 (62%) | 20 (32%) |  | 18 (69%) | 9 (39%) |  | 21 (72%) | 13 (42%) |  |
| yes | 22 (38%) | 42 (68%) |  | 8 (31%) | 14 (61%) |  | 8 (28%) | 18 (58%) |  |

NOTE: p value was calculated based on the univariable association analyses between each features and risk status of thymomas.

Abbreviations: SD, standard deviation; cm, centimeter; TPCL, ttumor perimeter contacted the lung; AA, acute angles between tumor lobulation and adjacent other structure; *, p value<0.1.

**Supplementary Table S2**. Risk factors for imaging features models

|  |  | **Model 1** |  |  |  | **Model 2** |  |
| --- | --- | --- | --- | --- | --- | --- | --- |
| **Intercept and Variable** | **β** | **Odds Ratio (95% CI)** | **P** |  | **β** | **Odds Ratio (95% CI)** | **P** |
| Intercept | -2.621 |  |  |  | -1.601 |  |  |
| contour | 1.186 | 3.275(1.817-5.905) | <0.001 |  | 0.859 | 3.343(1.887-5.924) | <0.001 |
| density | 1.365 | 3.914(1.621-9.449) | 0.002 |  | 1.221 | 3.342(1.465-7.622) | 0.004 |
| AA | 1.401 | 4.060(1.685-9.786) | 0.002 |  |  |  |  |
| C-index |  |  |  |  |  |  |  |
| Training cohort |  | 0.811(0.731-0.889) |  |  |  | 0.755(0.67-0.839) |  |
| Internal-validation cohort |  | 0.766(0.63-0.902) |  |  |  | 0.693(0.548-0.839) |  |
| External-validation cohort |  | 0.765(0.644-0.886) |  |  |  | 0.673(0.538-0.808) |  |

Abbreviations: OR, odds ratio; CI, confidence interval; AA, acute angles between tumor lobulations and adjacent other structure

**Supplementary Table S3**. Evaluation Of the imaging features models among cohorts

|  | **Model 1** | **Model 2** | **Chi-square** | **P** |
| --- | --- | --- | --- | --- |
| Training Cohort |  |  |  |  |
| AIC | 143.366 | 134.861 | 10.5 | 0.0012 |
| AUC | 0.8105 | 0.7551 | 2.232 | 0.1352 (Delong test) |
| Internal-validation Cohort |  |  |  |  |
| AIC | 66.001 | 64.354 | 3.65 | 0.0562 |
| AUC | 0.7659 | 0.6931 | 1.448 | 0.2289 (Delong test) |
| External-validation Cohort |  |  |  |  |
| AIC | 80.393 | 75.963 | 6.43 | 0.0112 |
| AUC | 0.7653 | 0.673 | 2.559 | 0.1097 (Delong test) |

Abbreviations: AIC, Akaike's information criterion; AUC, Area under curve.

**Supplementary** **Table S4**. Evaluation of individualized nomogram among cohorts

|  | Accuracy | Sensitivity | Specificity |
| --- | --- | --- | --- |
| Training Cohort | 77.50% | 74.19% | 81.03% |
| Internal-validation Cohort | 73.47% | 60.87% | 84.62% |
| External-validation Cohort | 70.00% | 70.97% | 68.97% |
